# Increased risk of psychiatric disorder in patients with hearing loss: a nationwide population-based cohort study

**DOI:** 10.1186/s12967-024-04992-4

**Published:** 2024-04-10

**Authors:** Qun-Yi Nian, Chun-An Cheng, Li-Hsiang Cheng, Yuan-Yung Lin, Chin-Hung Wang, Wu-Chien Chien, Yueng-Hsiang Chu, Cheng-Ping Shih, Chao-Yin Kuo, Hsin-Chien Chen, Jih-Chin Lee, Chi-Hsiang Chung, Wei-Chuan Shangkuan, Hung-Che Lin

**Affiliations:** 1 Department of Medicine, National Defense Medical Center, Taipei City, Taiwan; 2 Department of Neurology, Tri-Service General Hospital, National Defense Medical Center, Taipei City, Taiwan; 3 Department of Otolaryngology-Head and Neck Surgery, Tri-Service General Hospital, National Defense Medical Center, No. 325, Sec. 2, Chenggong Rd., Neihu Dist., 114202 Taipei City, Taiwan; 4 Department of Medical Research, Tri-Service General Hospital, National Defense Medical Center, Taipei City, Taiwan; 5 School of Public Health, National Defense Medical Center, Taipei City, Taiwan; 6 Graduate Institute of Life Sciences, National Defense Medical Center, Taipei City, Taiwan; 7 Taiwanese Injury Prevention and Safety Promotion Association, Taipei City, Taiwan; 8 Department of Otolaryngology-Head and Neck Surgery, Taipei City Hospital, Taipei City, Taiwan

**Keywords:** Psychiatric disorder, Hearing loss, Risk factor, Population-based retrospective cohort study

## Abstract

**Background:**

Hearing loss has been shown to be a risk factor for psychiatric disorders. In addition, long-term hearing loss is associated with increased hospitalization and mortality rates; however, the increased risk and duration of effect of hearing loss in combination with other chronic diseases on each psychiatric disorder are still not clearly defined. The purpose of this article is to clarify the risk of hearing loss for each disorder over time.

**Methods:**

This was a retrospective cohort study, and a national health insurance research database in Taiwan was utilized. All (n = 1,949,101) Taiwanese residents who had a medical visit between 2000 and 2015 were included. Patients with hearing loss and a comparative retrospective cohort were analyzed. Every subject was tracked individually from their index date to identify the subjects who later received a diagnosis of a psychiatric disorder. The Kaplan‒Meier method was used to analyze the cumulative incidence of psychiatric disorders. Cox regression analysis was performed to identify the risk of psychiatric disorders.

**Results:**

A total of 13,341 (15.42%) and 31,250 (9.03%) patients with and without hearing loss, respectively, were diagnosed with psychiatric disorders (P < 0.001). Multivariate analysis indicated that hearing loss significantly elevated the risk of psychiatric disorders (adjusted HR = 2.587, 95% CI 1.723–3.346, p < 0.001).

**Conclusion:**

Our findings indicate that patients with hearing loss are more likely to develop psychiatric disorders. Furthermore, the various psychiatric disorders are more likely to occur at different times. Our findings have important clinical implications, including a need for clinicians to implement early intervention for hearing loss and to pay close attention to patients’ psychological status.

*Trial registration* TSGHIRB No. E202216036.

**Supplementary Information:**

The online version contains supplementary material available at 10.1186/s12967-024-04992-4.

## Background

Hearing loss is a serious medical problem, and some types of hearing loss are irreversible [1]. According to the WHO, there are 538 million people with hearing loss worldwide [2], which is an average of one out of every 16 people. These patients face additional challenges in their daily lives, such as communication difficulties at work and at home, thus necessitating lifestyle changes. At the same time, people with hearing loss are at an increased risk of accidents (e.g., work-related, leisure-related) in their daily lives [3]. In clinical practice, most physicians assess patients in a relatively quiet environment, which is not comparable to patients' typical everyday surroundings; therefore, physicians cannot accurately assess the severity of hearing impairment in patients [4].

Psychiatric disorders have a tremendous impact on society. In addition to affecting the ability to work [5], they also have a negative impact on health [6]. However, if promptly managed, potential irreversible damage can be avoided [7–9]. Cognitive decline is also an issue in today's aging society. As cognitive decline progresses, some patients may develop dementia, and the subsequent medical care will become a great burden on the family, society, and medical institutions.

Long-term hearing loss not only increases the risk of depression, bipolar disorder, and cognitive decline but also increases hospitalization and mortality rates [10]. However, these articles only examine the relationship between a single disease and hearing loss. Furthermore, no research articles examine the relationship between hearing loss and multiple psychiatric disorders. Given the need for extensive population-based studies published on the risk of psychiatric disorders after hearing loss with a long-term follow-up, there is a clear gap in knowledge in this respect. Therefore, the purpose of this study is to analyze the risk of hearing loss for each psychiatric disorder by using large-scale study data from the National Health Insurance Research Database (NHIRD) in Taiwan.

## Methods

### Data source

In Taiwan, the National Health Insurance Program (NHIP) was launched on March 1, 1995, and currently provides over 99% coverage. The National Health Insurance Research Database (NHIRD) includes Taiwan's outpatient, emergency, and inpatient data. Under the policy, patients pay only a small portion of their medical costs, and hospitals rely on the data to request the remaining costs from insurance companies. If researchers want to use data from that database, they must pass a comprehensive review by a peer review committee. Research projects are approved by the Institutional Review Board at the TSGH. (TSGHIRB No. E202216036).

### Sample definition

In our study, we enrolled all patients with hearing impairment (ICD-9-CM: 389). Events with more than three visits, a diagnosed psychiatric disorder (ICD-9-CM: 290-319), and chronic disease were followed up. To minimize bias, we excluded patients with hearing impairment before 2000, events that occurred before follow-up, and patients of unknown sex. All study participants were followed up from the index date until the diagnosis of psychiatric disorders. Poor prognosis in this article was used as a collective term for psychiatric disorders, suicide or all-cause mortality, the details of which are listed in Additional file 1: Table S1.

To clarify the risk of psychiatric disorders for each age group, we divided the population into four groups: under 19 years old, 20 to 44 years old, 45 to 64 years old, and 65 years old and above. For the income classification (since premiums are correlated with income), we divided the same sample into three groups: those with premiums below 18,000 (NTD), those with premiums between 18,000 and 34,999, and those above 35,000 (NTD).

In addition, multisystem disease comorbidity may cause greater mental stress, so we listed the risks associated with systemic disease comorbidity. Based on the industrial structure and population density of each region, the degree of urbanization was classified into four levels; the regions were grouped into northern, central, southern, eastern, and outlying islands; and the hospitals were classified into regional hospitals, county hospitals and medical centers. Finally, to compare seasonal differences, we also categorized patients by the season when they were diagnosed with the disease.

**Fig. 1:**
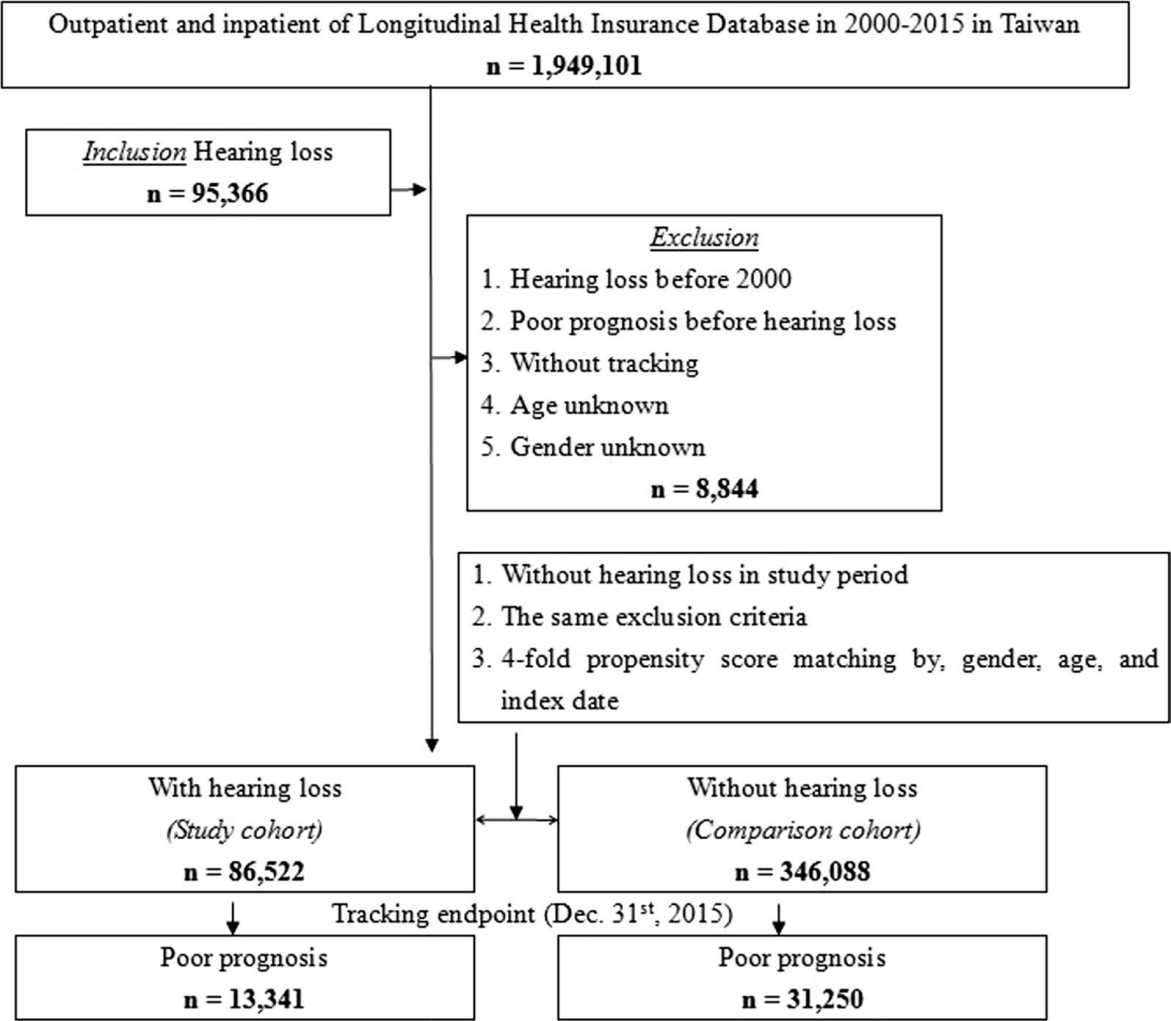
Flowchart of study sample selection from the National Health Insurance Research Data base in Taiwan

Figure 1 illustrates the design of our study, including the criteria for inclusion and exclusion. There were outpatient and inpatient data for 1,949,101 patients from January 1, 2000, to December 31, 2015, of whom 95,336 were diagnosed with hearing loss. Patients excluded were those with hearing loss prior to 2000 and those of unknown sex or age. In addition, those who had a psychiatric disorder before the diagnosis of hearing loss were also excluded. The final case group totaled 86,522 individuals. The same exclusion criteria were used for the control group as was used for the case group, and no hearing loss occurred during the study period. The control group, which included 346,088 individuals, was composed of four individuals per case-patient who were matched by index year, index month, sex, and age. These subjects were tracked through December 31, 2015 (Fig. 1).

### Statistical analysis

For categorical variables, frequencies and percentages were used, while for continuous variables, mean and standard deviation were used. To further compare the variations between the hearing loss and non-hearing loss groups at baseline and at the tracking endpoint, the X^2^ test and Mann‒Whitney U test (Wilcoxon rank-sum test) were utilized. The Kaplan–Meier method was used to estimate the difference in the risk of hearing loss between the study and observation groups, and a log-rank test was used to evaluate the difference between the groups in terms of the occurrence of cumulative hearing loss. The risk of psychiatric disorders was determined using multivariate Cox proportional hazards regression analysis, and the results were expressed as crude and adjusted hazard ratios (HRs) and 95% confidence intervals (CIs). The formula for the Cox regression analysis is as follows: for “crude HR”, Y (dependent variable) is poor prognosis, and X (independent variable) is each individual variable in the table (one Y corresponds to one X); for “adjusted HR”, Y (dependent variable) is poor prognosis, and X (independent variable) is all the variables in the table (one Y corresponds to multiple X). SAS 9.1 (SAS, Cary, NC) was used for the Schoenfeld residuals test. SPSS 22.0 (IBM, Armonk, NY) was used for all other statistical calculations, and a double-tailed p value of 0.05 was taken as the significant level.

## Results

The hearing loss group and the control group included 86,522 and 346,088 cases, respectively. Table 1 shows the demographic variables, psychiatric disorders, and other comorbidities for the hearing loss and control groups. There were no statistically significant differences in sex distribution between the hearing loss and control groups (p value: 0.999). In terms of psychiatric disorders, there was a significant difference in hearing loss compared to the control group (hearing loss group: 15.42%; control group: 9.03%; p value < 0.001). Regarding the demographic variables, in the age distribution, the majority of patients with hearing loss were younger than 19 years and older than 65 years (≦ 19: 33.63%; ≧ 65: 29.72%). The distribution of income was mostly concentrated in the group with a monthly income of less than NT$18,000 (< 18,000NT: 81%), but the distribution was relatively equal in terms of region and urbanization. The distribution of the population in terms of medical institutions increases from regional hospitals to medical centers. For other comorbidities, the hearing loss group had the highest rate in the injury group (injury: 21.84), followed by chronic diseases such as diabetes, hypertension, and kidney disease.

Table 2 shows the results of the Cox regression analysis for the risk of psychiatric disorders in the hearing loss group compared to the nonhearing-loss control group. The adjusted HR was 2.587 (95% CI 1.723–3.346, p < 0.001). The results showed that patients with hearing loss had a 2.587-fold higher risk of developing psychiatric disorders than those without hearing loss. Hearing loss had the highest adjusted HR relative to other items. We conducted a Schoenfeld residuals test based on the literature [11]. The global test for an adjusted HR showed a p value of 0.7941. The p value was not significant, indicating that the assumptions of Cox regression were satisfied [11]. We also performed an omnibus test of the Cox model for the adjusted HR, and the p value was significant, indicating that the model was reliable.

**Table 1:** Characteristics of study in the endpoint

Hearing loss	Total		With		Without		*P*
Variables	n	%	n	%	n	%	
Total	432,610		86,522	20.00	346,088	80.00	
Poor prognosis							< *0.001*
Without	388,019	89.69	73,181	84.58	314,838	90.97	
With	44,591	10.31	13,341	15.42	31,250	9.03	
Sex							0.999
Male	222,560	51.45	44,512	51.45	178,048	51.45	
Female	210,050	48.55	42,010	48.55	168,040	48.55	
Age (years)	45.44 ± 19.59		43.33 ± 18.92		45.97 ± 19.76		< *0.001*
Age groups (years)							< *0.001*
≦ 19	139,499	32.25	29,101	33.63	110,398	31.90	
20–44	72,821	16.83	14,501	16.76	58,320	16.85	
45–64	87,506	20.23	17,203	19.88	70,303	20.31	
≧ 65	132,784	30.69	25,717	29.72	107,067	30.94	
Insured premium (NT$)							< *0.001*
< 18,000	349,095	80.70	70,124	81.05	278,971	80.61	
18,000–34,999	46,022	10.64	11,012	12.73	35,010	10.12	
≧ 35,000	37,493	8.67	5386	6.23	32,107	9.28	
DM							< *0.001*
Without	380,916	88.05	75,401	87.15	305,515	88.28	
With	51,694	11.95	11,121	12.85	40,573	11.72	
HTN							< *0.001*
Without	377,460	87.25	73,024	84.40	304,436	87.96	
With	55,150	12.75	13,498	15.60	41,652	12.04	
Renal disease							< *0.001*
Without	375,379	86.77	72,192	83.44	303,187	87.60	
With	57,231	13.23	14,330	16.56	42,901	12.40	
Hyperlipidemia							< *0.001*
Without	404,343	93.47	79,157	91.49	325,186	93.96	
With	28,267	6.53	7365	8.51	20,902	6.04	
Thyrotoxicosis							0.872
Without	407,640	94.23	81,453	94.14	326,187	94.25	
With	24,970	5.77	5069	5.86	19,901	5.75	
Septicemia							0.837
Without	428,301	99.00	85,650	98.99	342,651	99.01	
With	4309	1.00	872	1.01	3,437	0.99	
Pneumonia							< *0.001*
Without	392,434	90.71	77,510	89.58	314,924	91.00	
With	40,176	9.29	9012	10.42	31,164	9.00	
CLD							< *0.001*
Without	390,370	90.24	77,484	89.55	312,886	90.41	
With	42,240	9.76	9038	10.45	33,202	9.59	
Injury							< *0.001*
Without	372,410	86.08	67,625	78.16	304,785	88.07	
With	60,200	13.92	18,897	21.84	41,303	11.93	
Tumor							0.422
Without	422,302	97.62	84,300	97.43	338,002	97.66	
With	10,308	2.38	2222	2.57	8,086	2.34	
Season							0.084
Spring	102,754	23.75	20,875	24.13	81,879	23.66	
Summer	107,037	24.74	21,065	24.35	85,972	24.84	
Autumn	113,544	26.25	22,872	26.43	90,672	26.20	
Winter	109,275	25.26	21,710	25.09	87,565	25.30	
Location							< *0.001*
Northern Taiwan	122,620	28.34	25,008	28.90	97,612	28.20	
Central Taiwan	120,130	27.77	23,232	26.85	96,898	28.00	
Southern Taiwan	119,793	27.69	24,121	27.88	95,672	27.64	
Eastern Taiwan	47,967	11.09	9855	11.39	38,112	11.01	
Outlying islands	22,100	5.11	4306	4.98	17,794	5.14	
Urbanization level							< *0.001*
1 (the highest)	123,646	28.58	24,952	28.84	98,694	28.52	
2	129,123	29.85	25,771	29.79	103,352	29.86	
3	86,125	19.91	16,101	18.61	70,024	20.23	
4 (the lowest)	93,716	21.66	19,698	22.77	74,018	21.39	
Level of care							< *0.001*
Hospital center	131,624	30.43	31,652	36.58	99,972	28.89	
Regional hospital	161,052	37.23	30,042	34.72	131,010	37.85	
Local hospital	139,934	32.35	24,828	28.70	115,106	33.26	

p: Chi-square test on category variables and Mann–Whitney U-test on continue variables

Table 3 compares “hearing loss or not” with the other variables in the reference table. Regarding the demographic variables, the risk of psychiatric disorders increased with age in the hearing loss group compared to the control group, and a similar pattern was observed for the urbanization and level of medical facility categories. In the winter subgroup of the hearing loss group, the risk of psychiatric disorders was 2.62-fold higher than that of the nonhearing-loss group. In the comorbidity subgroup, having other comorbidities slightly increased the risk of psychiatric disorders in the hearing loss group. For example, in the group without diabetes, the hearing loss group had a 2.57-fold risk of psychiatric disorders in the nonhearing-loss group, and their risk increased to 2.66-fold if they had diabetes.

**Table 2:** Factors of poor prognosis by using Cox regression

Variables	Crude HR	Lower 95% CI	Upper 95% CI	*P*	Adjusted HR	Lower 95% CI	Upper 95% CI	*P*
Hearing loss								
Without	Reference				Reference			
With	2.994	2.097	3.787	< *0.001*	2.587	1.723	3.346	< *0.001*
Sex								
Male	1.562	1.146	1.897	< *0.001*	1.340	1.025	1.798	*0.025*
Female	Reference				Reference			
Age groups (years)								
≦ 19	Reference				Reference			
20–44	1.597	1.344	1.825	< *0.001*	1.444	1.102	1.789	< *0.001*
45–64	2.234	1.602	2.570	< *0.001*	2.101	1.527	2.482	< *0.001*
≧ 65	2.456	1.842	2.677	< *0.001*	2.301	1.793	2.571	< *0.001*
Insured premium (NT$)								
< 18,000	Reference				Reference			
18,000–34,999	0.897	0.672	1.795	0.312	0.721	0.558	1.422	0.486
≧ 35,000	0.786	0.533	1.564	0.467	0.611	0.402	1.303	0.597
DM								
Without	Reference				Reference			
With	2.012	1.450	2.795	< *0.001*	1.861	1.303	2.672	< *0.001*
HTN								
Without	Reference				Reference			
With	2.370	1.187	3.301	< *0.001*	2.287	1.104	3.010	< *0.001*
Renal disease								
Without	Reference				Reference			
With	2.165	1.352	2.779	< *0.001*	2.006	1.254	2.650	< *0.001*
Hyperlipidemia								
Without	Reference				Reference			
With	1.786	1.256	2.352	< *0.001*	1.701	1.124	2.245	< *0.001*
Thyrotoxicosis								
Without	Reference				Reference			
With	1.568	1.056	1.896	< *0.001*	1.332	0.894	1.672	0.150
Septicemia								
Without	Reference				Reference			
With	1.898	1.265	2.442	< *0.001*	1.562	1.059	2.204	*0.001*
Pneumonia								
Without	Reference				Reference			
With	1.562	1.131	1.886	< *0.001*	1.482	1.101	1.782	
CLD								
Without	Reference				Reference			
With	2.465	1.562	2.976	< *0.001*	2.323	1.397	2.864	< *0.001*
Injury								
Without	Reference				Reference			
With	2.551	1.334	3.792	< *0.001*	2.154	1.202	2.970	< *0.001*
Tumor								
Without	Reference				Reference			
With	2.865	1.562	3.971	< *0.001*	2.229	1.488	3.125	< *0.001*
Season								
Spring	Reference				Reference			
Summer	1.350	1.145	1.551	< *0.001*	1.182	0.899	1.443	0.120
Autumn	1.577	1.301	1.803	< *0.001*	1.297	1.030	1.625	*0.021*
Winter	1.702	1.565	1.976	< *0.001*	1.488	1.206	1.896	< *0.001*
Location					Multicollinearity with urbanization level			
Northern Taiwan	Reference				Multicollinearity with urbanization level			
Central Taiwan	0.862	0.452	1.452	0.592	Multicollinearity with urbanization level			
Southern Taiwan	1.067	0.651	1.570	0.389	Multicollinearity with urbanization level			
Eastern Taiwan	0.675	0.202	1.265	0.887	Multicollinearity with urbanization level			
Outlying islands	1.124	0.756	1.797	0.311	Multicollinearity with urbanization level			
Urbanization level								
1 (the highest)	1.986	1.399	2.706	< *0.001*	1.906	1.284	2.669	< *0.001*
2	1.911	1.382	2.689	< *0.001*	1.896	1.277	2.652	< *0.001*
3	1.543	1.165	2.244	< *0.001*	1.401	1.073	2.184	*0.001*
4 (the lowest)	Reference				Reference			
Level of care								
Hospital center	2.118	1.601	2.798	< *0.001*	1.996	1.485	2.501	< *0.001*
Regional hospital	1.870	1.375	2.505	< *0.001*	1.756	1.246	2.334	< *0.001*
Local hospital	Reference				Reference			

*HR* hazard ratio, *CI* confidence interval, *Adjusted HR* Adjusted variables listed in the table

Adjusted HR model, Schoenfeld's global test: p = 0.7941; Omnibus test: p < 0.001

Additional file 2: Table S3 classifies patients with hearing loss. There are two groups, the first being diagnoses made by otolaryngologists and the second being diagnoses made by other specialists. Other specialists included those in the fields of family medicine, emergency medicine and pediatrics. The two groups with hearing loss both have a similar chance of having psychiatric disorders.

Table 4 presents the subgroups of psychiatric disorders, and we list the common diagnoses, such as depression, bipolar disorder, or PTSD.

**Table﻿ 3:** Factors of poor prognosis stratified by variables listed in the table by using Cox regression and Bonferroni correction for multiple comparisons

Hearing loss	With			Without *(Reference)*			With* vs.* Without *(Reference)*			
Strarified	Events	PYs	Rate (per 10^5^ PYs)	Events	PYs	Rate (per 10^5^ PYs)	Adjusted HR	Lower 95% CI	Upper 95% CI	*P*
Total	13,341	828,013.26	1611.21	31,250	3,377,815.10	925.15	2.587	1.723	3.346	< *0.001*
Sex										
Male	6869	425,978.31	1612.52	16,074	1,773,776.21	906.20	2.643	1.760	3.419	< *0.001*
Female	6472	402,034.95	1609.81	15,176	1,604,038.89	946.11	2.528	1.683	3.269	< *0.001*
Age groups (years)										
≦ 19	4385	278,495.11	1574.53	9864	1,077,483.29	915.47	2.555	1.702	3.304	< *0.001*
20–44	2206	138,774.06	1589.63	5220	569,202.14	917.07	2.575	1.715	3.330	< *0.001*
45–64	2672	164,632.18	1623.01	6345	686,156.01	924.72	2.607	1.736	3.372	< *0.001*
≧ 65	4078	246,111.91	1656.97	9821	1,044,973.66	939.83	2.619	1.744	3.387	< *0.001*
Insured premium (NT$)										
< 18,000	10,813	671,084.29	1611.27	25,151	2,722,753.90	923.73	2.591	1.726	3.351	< *0.001*
18,000–34,999	1699	105,385.07	1612.18	3154	346,972.24	909.01	2.635	1.755	3.408	< *0.001*
≧ 35,000	829	51,543.90	1608.34	2945	308,088.96	955.89	2.499	1.665	3.233	< *0.001*
DM										
Without	11,516	721,585.98	1595.93	27,461	2,981,822.99	920.95	2.574	1.714	3.329	< *0.001*
With	1825	106,427.28	1714.79	3789	395,992.11	956.84	2.662	1.773	3.443	< *0.001*
HTN										
Without	11,152	698,837.66	1595.79	27,373	2,971,291.36	921.25	2.573	1.714	3.328	< *0.001*
With	2189	129,175.60	1694.59	3877	406,523.74	953.70	2.639	1.758	3.414	< *0.001*
Renal disease										
Without	11,074	690,875.90	1602.89	27,349	2,959,090.13	924.24	2.576	1.716	3.332	< *0.001*
With	2267	137,137.36	1653.09	3901	418,724.97	931.64	2.636	1.755	3.409	< *0.001*
Hyperlipidemia										
Without	12,091	757,529.96	1596.11	29,353	3,173,780.81	924.86	2.564	1.707	3.316	< *0.001*
With	1250	70,483.30	1773.47	1897	204,034.29	929.75	2.833	1.887	3.665	< *0.001*
Thyrotoxicosis										
Without	12,544	779,506.53	1609.22	29,447	3,183,579.89	924.97	2.584	1.721	3.343	< *0.001*
With	797	48,506.73	1643.07	1803	194,235.21	928.26	2.629	1.751	3.401	< *0.001*
Septicemia										
Without	13,155	819,668.15	1604.92	30,916	3,344,269.97	924.45	2.579	1.718	3.335	< *0.001*
With	186	8,345.11	2228.85	334	33,545.13	995.67	3.325	2.215	4.301	< *0.001*
Pneumonia										
Without	11,916	741,768.71	1606.43	28,425	3,073,654.34	924.79	2.580	1.719	3.337	< *0.001*
With	1425	86,244.55	1652.28	2825	304,160.76	928.79	2.643	1.760	3.418	< *0.001*
CLD										
Without	11,905	741,518.50	1605.49	28,250	3,053,764.72	925.09	2.578	1.717	3.334	< *0.001*
With	1436	86,494.76	1660.22	3000	324,050.38	925.78	2.664	1.774	3.445	< *0.001*
Injury										
Without	10,306	647,163.34	1592.49	27,506	2,973,983.95	924.89	2.558	1.703	3.308	< *0.001*
With	3035	180,849.92	1678.19	3744	403,831.15	927.12	2.689	1.791	3.478	< *0.001*
Tumor										
Without	12,961	806,748.42	1606.57	30,515	3,298,895.83	925.01	2.580	1.718	3.337	< *0.001*
With	380	21,264.84	1786.99	735	78,919.27	931.33	2.850	1.898	3.686	< *0.001*
Season										
Spring	3114	199,773.22	1558.77	7246	799,125.13	906.74	2.554	1.701	3.303	< *0.001*
Summer	3185	201,597.46	1579.88	7644	838,970.17	911.12	2.576	1.716	3.331	< *0.001*
Autumn	3536	218,840.23	1615.79	8188	884,957.26	925.24	2.594	1.728	3.355	< *0.001*
Winter	3506	207,802.35	1687.18	8172	854,762.54	956.05	2.621	1.746	3.391	< *0.001*
Urbanization level										
1 (the highest)	3975	238,798.65	1664.58	8925	963,525.17	926.29	2.669	1.778	3.453	< *0.001*
2	4011	246,635.11	1626.29	9334	1,008,725.27	925.33	2.611	1.739	3.377	< *0.001*
3	2422	154,086.98	1571.84	6322	683,434.10	925.03	2.524	1.681	3.265	< *0.001*
4 (the lowest)	2933	188,492.52	1556.03	6669	722,130.56	923.52	2.503	1.667	3.237	< *0.001*
Level of care										
Hospital center	4975	302,908.15	1642.41	9046	975,276.31	927.53	2.630	1.752	3.402	< *0.001*
Regional hospital	4633	287,501.34	1611.47	11,845	1,278,985.60	926.12	2.585	1.721	3.343	< *0.001*
Local hospital	3733	237,603.77	1571.10	10,359	1,123,553.19	921.99	2.531	1.686	3.274	< *0.001*

*PYs* Person-years, *Adjusted HR* Adjusted Hazard ratio: Adjusted for the variables listed in Table 3.; *CI* confidence interval

Three models are presented: the first model is without restrictions, and the remaining two models excluded patients with hearing loss who are diagnosed with a psychiatric disorder within the first year of follow-up and within the first five years of follow-up. This was used to differentiate the impact of each period, as shown in Additional file 3: Table S4.

The risk of PTSD/ASD was highest in the first two groups (HR ratio: 3.371 and 3.253) but was not statistically significant in the third group (p value: 0.101), which meant that it had a medium-term impact. The second highest risk was for depression and anxiety, which was statistically significant in all groups.

**Table 4:** Factors of poor prognosis subgroups by using Cox regression and Bonferroni correction for multiple comparisons

Sensitivity test	Hearing loss	With			Without *(Reference)*			With vs. without *(Reference)*			
	Poor prognosis subgroups	Events	PYs	Rate (per 10^5^ PYs)	Events	PYs	Rate (per 10^5^ PYs)	Adjusted HR	Lower 95% CI	Upper 95% CI	*P*
Overall	Overall	13,341	828,013.26	1611.21	31,250	3,377,815.10	925.15	2.587	1.723	3.346	< *0.001*
	Mental disoredes	11,084	828,013.26	1338.63	24,874	3,377,815.10	736.39	2.701	1.796	3.495	< *0.001*
	Anxiety	2677	828,013.26	323.30	5211	3,377,815.10	154.27	3.114	2.076	4.025	< *0.001*
	Depression	3167	828,013.26	382.48	5955	3,377,815.10	176.30	3.225	2.146	4.168	< *0.001*
	Bipolar	1523	828,013.26	183.93	4112	3,377,815.10	121.74	2.245	1.496	2.904	< *0.001*
	Sleep disorders	1912	828,013.26	230.91	4512	3,377,815.10	133.58	2.568	1.710	3.323	< *0.001*
	PTSD/ASD	230	828,013.26	27.78	413	3,377,815.10	12.23	3.371	2.248	4.356	< *0.001*
	Dementia	188	828,013.26	22.70	522	3,377,815.10	15.45	2.182	1.457	2.833	< *0.001*
	Eating disorders	311	828,013.26	37.56	811	3,377,815.10	24.01	2.234	1.549	3.007	< *0.001*
	SRD	296	828,013.26	35.75	823	3,377,815.10	24.36	2.179	1.452	2.820	< *0.001*
	Psychotic disorders	270	828,013.26	32.61	772	3,377,815.10	22.86	2.118	1.412	2.740	< *0.001*
	Autism	58	828,013.26	7.00	201	3,377,815.10	5.95	1.749	1.167	2.263	< *0.001*
	Other mental disorders	452	828,013.26	54.59	1542	3,377,815.10	45.65	1.775	1.183	2.297	< *0.001*
	Suicide	156	828,013.26	18.84	597	3,377,815.10	17.67	1.583	1.048	2.049	*0.002*
	All-caused mortality	2101	828,013.26	253.74	5779	3,377,815.10	171.09	2.204	1.467	2.879	< *0.001*
In the first year excluded	Overall	12,007	776,918.33	1545.46	28,108	3,166,702.49	887.61	2.586	1.723	3.345	< *0.001*
	Mental disoredes	9977	776,918.33	1284.18	22,388	3,166,702.49	706.98	2.698	1.797	3.490	< *0.001*
	Anxiety	2408	776,918.33	309.94	4688	3,166,702.49	148.04	3.110	2.071	4.022	< *0.001*
	Depression	2851	776,918.33	366.96	5354	3,166,702.49	169.07	3.224	2.147	4.170	< *0.001*
	Bipolar	1371	776,918.33	176.47	3697	3,166,702.49	116.75	2.245	1.495	2.904	< *0.001*
	Sleep disorders	1724	776,918.33	221.90	4058	3,166,702.49	128.15	2.572	1.713	3.327	< *0.001*
	PTSD/ASD	209	776,918.33	26.90	389	3,166,702.49	12.28	3.253	2.167	4.207	< *0.001*
	Dementia	169	776,918.33	21.75	470	3,166,702.49	14.84	2.177	1.450	2.816	< *0.001*
	Eating disorders	280	776,918.33	36.04	729	3,166,702.49	23.02	2.326	1.549	3.008	< *0.001*
	SRD	266	776,918.33	34.24	744	3,166,702.49	23.49	2.165	1.442	2.800	< *0.001*
	Psychotic disorders	243	776,918.33	31.28	691	3,166,702.49	21.82	2.129	1.418	2.754	< *0.001*
	Autism	52	776,918.33	6.69	183	3,166,702.49	5.78	1.720	1.146	2.225	< *0.001*
	Other mental disorders	404	776,918.33	52.00	1385	3,166,702.49	43.74	1.766	1.176	2.284	< *0.001*
	Suicide	131	776,918.33	16.86	519	3,166,702.49	16.39	1.528	1.018	1.977	*0.033*
	All-caused mortality	1899	776,918.33	244.43	5201	3,166,702.49	164.24	2.211	1.472	2.859	< *0.001*
In the first 5 years excluded	Overall	6657	568,149.17	1171.70	17,675	2,322,248.05	761.12	2.287	1.523	2.958	< *0.001*
	Mental disoredes	5529	568,149.17	973.16	14,068	2,322,248.05	605.79	2.386	1.589	3.086	< *0.001*
	Anxiety	1366	568,149.17	240.43	2947	2,322,248.05	126.90	2.814	1.874	3.640	< *0.001*
	Depression	1611	568,149.17	283.55	3369	2,322,248.05	145.07	2.903	1.934	3.755	< *0.001*
	Bipolar	760	568,149.17	133.77	2326	2,322,248.05	100.16	1.984	1.321	2.566	< *0.001*
	Sleep disorders	954	568,149.17	167.91	2551	2,322,248.05	109.85	2.271	1.512	2.937	< *0.001*
	PTSD / ASD	55	568,149.17	9.68	245	2,322,248.05	10.55	1.363	0.908	1.763	0.101
	Dementia	74	568,149.17	13.02	303	2,322,248.05	13.05	1.483	0.988	1.918	0.072
	Eating disorders	155	568,149.17	27.28	459	2,322,248.05	19.77	2.050	1.366	2.652	< *0.001*
	SRD	158	568,149.17	27.81	461	2,322,248.05	19.85	2.081	1.386	2.691	< *0.001*
	Psychotic disorders	135	568,149.17	23.76	432	2,322,248.05	18.60	1.897	1.264	2.454	< *0.001*
	Autism	35	568,149.17	6.16	111	2,322,248.05	4.78	1.914	1.275	2.476	< *0.001*
	Other mental disorders	226	568,149.17	39.78	864	2,322,248.05	37.21	1.588	1.058	2.054	< *0.001*
	Suicide	80	568,149.17	14.01	330	2,322,248.05	14.21	1.465	0.976	1.895	0.089
	All-caused mortality	1048	568,149.17	184.52	3277	2,322,248.05	141.11	1.942	1.294	2.512	< *0.001*

*PYs* Person-years; *Adjusted HR* Adjusted Hazard ratio: Adjusted for the variables listed in Table 3; *CI* confidence interval

## Discussion

This is the first pilot study to define explicit risk associations between hearing loss and psychiatric disorders utilizing a large national population. Based on subgroup analysis, we also identified the most common psychiatric disorders caused by hearing loss, with the highest to lowest rates ranked as post-traumatic stress syndrome (PTSD), depression, and anxiety disorders. In this study, we provide more detailed information on the risk of psychiatric disorders in patients with hearing loss. For health care professionals, better health care plans can be developed, and for public health workers, more comprehensive social welfare programs can be established to reduce overall health care costs.

In Table 2, we list the risk factors for psychiatric disorders and calculate the hazard ratio. Compared to other comorbidities and demographic variables, hearing loss had the highest risk of developing psychiatric disorders. In Table 3, we compare the effects of different conditions on the hearing loss group. The risk of psychiatric disorders in the hearing loss group was found to increase with age. The elderly group’s hazard ratio was greater than that of the younger group (2.610 and 2.555). This could be related to other comorbidities. Systemic diseases are already a stressor for psychiatric disorders [12], and hearing loss is also considered a stressor [4], so a higher risk can be expected when both disorders are present, and this hypothesis is confirmed by our statistical data. In addition, the effect of seasonal changes on patients with hearing loss can be explained by seasonal affective disorder. Psychiatric disorders are more likely to develop in the winter [13], and this is no exception in the hearing loss community. In Additional file 2: Table S3, an additional test was conducted due to concerns about possible discrepancies in the diagnosis of hearing loss between doctors in different specialties, but the results did not appear to be significantly different.

In compiling the data, we found a phenomenon: the more urbanized the patient was, the higher the risk of developing psychiatric disorders. Another study supports our findings [14]. We believe this is because urban living requires more effective communication. This means that patients with hearing loss need to be fully attentive to every message they receive, which inevitably stresses patients with hearing loss. In Taiwan, most medical centers are in highly urbanized areas, where they receive more patients with hearing loss and more complex diseases. As a result, patients at medical centers have a higher risk of developing psychiatric disorders after follow-up visits (adjusted HR = 2.630). Patients in medical centers usually have more complicated medical problems.

Scholars have confirmed that hearing loss is a risk factor for psychiatric disorders [4] and that it also contributes to cognitive decline [15]. We make inferences about this observation, which can be divided into two main categories: psychological and physical. Psychologically, hearing loss is stressful for patients who need to compensate for their hearing deficits by reading lips. Furthermore, since the COVID-19 outbreak, the public has been wearing masks, which makes it more difficult for patients with hearing loss to understand the verbal communication of others [16]. Despite the fact that hearing aids have been shown to be effective in lowering the incidence of psychiatric disorders, there is still a gap when compared to the general population, most likely due to the noticeable appearance of hearing aids and poor sound recognition [17]. Additionally, when patients with hearing loss suffer from psychiatric disorders, they are unable to communicate effectively with physicians in psychiatric outpatient clinics because most physicians are not proficient in sign language. Therefore, a third person who understands sign language is usually needed to relay the message, but this may distort the message and make it difficult for the physician to have a more accurate handle on the condition. Physiologically, studies have shown that when a patient suffers from hearing loss, there are changes in the volume of relevant areas of the brain, causing the surrounding brain tissue to be affected and eventually causing cognitive impairment [18, 19]. This progression is very slow, and by the time psychiatric symptoms appear, a significant amount of brain tissue has already atrophied.

**Fig. 2:**
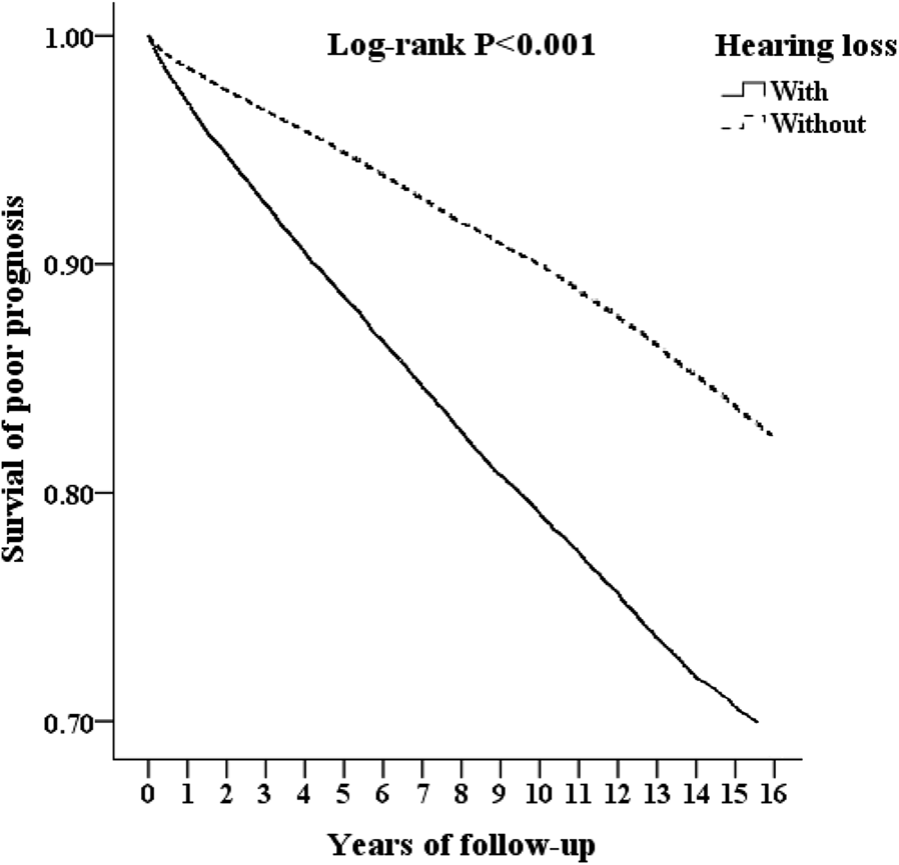
Kaplan‒Meier analysis of poor prognosis stratified by hearing loss with the log-rank test. Kaplan‒Meier analysis showed that patients with hearing loss had a significantly higher rate of psychiatric disorders than matched controls (log test p < 0.001)

The Kaplan‒Meier method for long-term follow-up of cumulative risk of psychiatric disorders had a statistically significant log-rank (p < 0.001) (Fig. 2). In our study, patients with hearing loss may have developed symptoms slowly over time, in addition to psychiatric symptoms in the short term.

In our event subgroup analysis, we found that patients had the highest risk of ASD and PTSD within the first five years of diagnosis (adjusted HR = 3.371). We also found other articles that supported our data and hypothesis [20] that acquired hearing loss may result in ASD or PTSD symptoms, as well as underlying stressful pre-existing relationship breakdowns, job failures, or an inability to adapt. In the long term, the risk of anxiety and depression is more than three times greater in these patients. Charlene J. Crump et al. showed that patients with hearing loss were often unable to communicate effectively with their psychiatrists during long-term local follow-up visits, which then affected their judgment and treatment, leading to a worsening of their disease [21]. Age, chronic disease, sensory stimulation loss, e.g., hearing loss, and other variables can all contribute to cognitive decline. The use of cochlear implants [22] or hearing aids [23] can significantly decrease cognitive decline, improving the elderly's capacity for self-care [24] and lowering the cost of long-term care services.

In addition to evaluating the use of hearing aids or cochlear implants, we recommend that clinicians working with patients who are diagnosed with hearing loss briefly assess the patient’s cognitive and mental status at the outpatient follow-up visit. If psychiatric disorders are suspected, early consultation with a neurologist or psychiatrist for evaluation and treatment is indicated. Some hearing losses, such as age-related hearing loss, progress over time [25]. This means that the sooner a psychiatrist is involved, the better the patient's chances of communicating effectively with him or her.

More importantly, in the public health context, this study provides a more accurate risk ratio than other studies, which can be used to predict the number of patients with psychiatric disorders in the future, as well as to assess the budget for subsidies, such as reducing the cost of hearing aids, cochlear implants, auditory brainstem implants, or sign language education. The strength of this study is mainly in the large sample size obtained from our national database to statistically determine the relative risk, as no previous large-scale study has statistically determined the risk ratio of hearing loss for each psychiatric disorder. In addition, the long-term follow-up made it possible to analyze short-, medium-, and long-term effects at the same time.

However, there are still limitations to this study. First, there is no detailed information on the severity of hearing loss. There is no way to specify the progress of each patient with psychiatric disorders. In other words, we cannot know whether hearing loss is a key incident in the development of psychiatric disorders. It may be that a patient has symptoms of a psychiatric disorder prior to hearing loss but has not been diagnosed. However, this can be minimized by extending the follow-up years to exclude the first year or the first 5 years. Statistically significant differences were seen for most of the subgroups (p value < 0.001). Second, these tables do not include medications other than those used for hearing loss. For example, proton pump inhibitors can lead to depression in some patients with specific physical conditions [26], but we believe that this has very little impact on the results of the study because the percentage of people is small and the condition can be cured by changing the medication. Third, we were unable to perform an accurate sensitivity test to diagnose hearing loss. However, we conducted an additional test, shown in Additional file 2: Table S3, to compare patients with hearing loss diagnosed by otolaryngologists and other specialists who had a poor prognosis when followed up over time. The results were similar. Fourth, patients with each type of psychiatric disorder may have different backgrounds. Therefore, the impact of hearing loss on the development of each psychiatric disorder may be different. The potential mechanisms underlying individual responses remain to be further examined on a large scale.

## Conclusions

This study with long-term follow-up demonstrated that hearing loss significantly increases the risk of developing psychiatric disorders and that the risk increases with age. Patients with hearing loss who also have other comorbidities are at a higher risk for psychiatric disorders. For this reason, we recommend that clinicians not overlook the clinical importance of these relationships. During outpatient hearing examinations, it is recommended to additionally assess the patient's cognitive and mental status and refer patients with low status to neurologists or psychiatrists.

### Supplementary Information

**Additional file 1: Table S1.** Abbreviations, ICD-9-CM codes, and definitions.

**Additional file 2: Table S3.** Factors of poor prognosis among different diagnosed department of hearing loss by using Cox regression and Bonferroni correction for multiple comparisons.

**Additional file 3: Table S4.** Analysis of whether events had a short-, medium-, or long-term impact.

## Data Availability

All the data underlying the present study are from the National Health Insurance Research Database (NHIRD). Researchers can obtain the data through formal application to the Ministry of Health and Welfare, Taiwan.

## Abbreviations

HR: Hazard ratio
CI: Confidence interval
PTSD: Posttraumatic stress disorder
CLD: Chronic liver disease
ASD: Acute stress disorder
SRD: Substance-related disorder
DM: Diabetes mellitus
HTN: Hypertension
PYs: Person-years
